# The Electro-Magnet in Ophthalmic Surgery

**Published:** 1889-11-02

**Authors:** C. S. Jeaffreson

**Affiliations:** Senior Surgeon to the Northumberland, Durham, and Newcastle Infirmary for Diseases of the Eye


### Editor's Letter-Box.

[Our Correspondents are reminded that prolixity is a great bar to publication, and that brevity of style and conciseness of statement greatly facilitate early insertion.]

### THE ELECTROMAGNET IN OPHTHALMIC SURGERY.

My attention has been called to some remarks in your issue of October 19, concerning a letter of Mr. Snell's "On the use of the Electromagnet in Ophthalmic Surgery." In those remarks you say a Mr. Snell observes that he sees no mention of this plan in a recent clinical lecture "On the Removal of Foreign Bodies from the Eye." As the author of the lecture referred to, you will perhaps allow me to correct this very glaring misstatement, for, not only is the electromagnet referred to more than once, but the absolute details of a case in which it was used for the removal of a foreign body are given in detail. Mr. Snell's remarks have no doubt led you, as they have others, into this error, which I hope you will correct in your next issue.

C. S. Jeaffreson,
Senior Surgeon to the Northumberland, Durham, and Newcastle Infirmary for Diseases of the Eye.

